# Similarly Torn, Differentially Shorn? The Experience and Management of Conflict between Multiple Roles, Relationships, and Social Categories

**DOI:** 10.3389/fpsyg.2017.01732

**Published:** 2017-10-05

**Authors:** Janelle M. Jones, Michaela Hynie

**Affiliations:** ^1^Department of Biological and Experimental Psychology, School of Biological and Chemical Sciences, Queen Mary University of London, London, United Kingdom; ^2^Department of Psychology, York University, Toronto, ON, Canada

**Keywords:** multiple identities, identity conflict, conflict management strategies, roles, relationships, social categories

## Abstract

In three studies we examined the experience and management of conflict between different types of multiple identities. Participants described a conflict between pairs of role, relational, or social identities before rating the experience (i.e., magnitude, stress, and growth) and management of conflict on a newly developed scale assessing four strategies: *reconciliation*, where identities are integrated, *realignment*, where one identity is chosen over another, *retreat*, where both identities are avoided, and *reflection*, where fit (with others, situation) determines identity selection. In general, the types of identities mattered for conflict management but not its experience: Magnitude and growth did not differ, however, stress was greater for role identity conflicts (*Study 3 only*) and participants endorsed the use of more realignment for role conflicts (*Study 2*) and more retreat for relational conflicts (*Study 3*) relative to other types of identity conflicts. Furthermore, findings suggested that the perceived flexibility of identities, not their importance or valence, were associated with realignment and retreat for roles and with retreat for relationships. Experiencing conflicts between multiple identities leaves people similarly torn, but multiple roles and relationships may be differentially shorn to manage conflict.

## Introduction

Student. Friend. British. These are some of the roles, relationships and social categories from which individuals can derive a sense of identity – knowledge of who they are, how they should act, and/or their place in the world (Tajfel, 1981; Tajfel and Turner, 1986; Turner et al., 1994; Deaux et al., 1995). These *multiple identities* are important ways that individuals come to understand themselves and others. Indeed, a considerable body of theoretical and empirical work on multiple identities has shed light on the ways that individuals perceive multiply categorizable targets and respond to (mis-) categorizations by others, as well as the implications of multiple identities for individuals’ cognition, well-being, and interactions (see Kang and Bodenhausen, 2015 for a comprehensive review). However, our understanding of how individuals experience and manage multiple identities within themselves is still in its infancy. Existing work has focused on the tension between multiple cultural identities (i.e., biculturalism; Benet-Martínez et al., 2002;

Benet-Martínez and Haritatos, 2005) or multiple racial identities (Shih and Sanchez, 2005; Sanchez et al., 2009). Yet, as highlighted above, social categories such as culture and race reflect only one possible type of identity. The extent to which other types of identities such as roles and/or relationships result in tension requires further scrutiny. Moreover, given people’s increasing awareness and adoption of multiple identities across contexts and time (e.g., Hodges and Park, 2013; Goclowska and Crisp, 2014), the nature of any tension experienced and how it is managed for different types of identities are important empirical questions.

To begin to address these issues, the present research conducted three studies on the experience and management of *identity conflict*, a perceived intrapersonal tension, discrepancy or interference, between at least two identities. In particular we focused on identity conflicts between pairs of roles, relationships, and social categories – key dimensions on which individuals base their identities (Baumeister et al., 1985; Deaux et al., 1995; Benet-Martínez et al., 2002; Settles, 2004; Benet-Martínez and Haritatos, 2005; Hirsh and Kang, 2016). We assessed the experience of conflict between multiple role, relational, and social identities by focusing on perceptions of conflict magnitude and associated stress and growth. Drawing from psychological, sociological and cultural theories on multiple identities (Baumeister et al., 1985; Berry, 1997, 2005; Roccas and Brewer, 2002; Burke, 2003, 2006; Goclowska and Crisp, 2014; Amiot et al., 2015) we developed a scale to assess the management of conflict between multiple identities. We also considered whether the characteristics associated with role, relational, and social identities, namely flexibility, valence, and importance (Deaux et al., 1995; Settles, 2004; Brook et al., 2008; Rabinovich and Morton, 2016) might influence the experience and/or management of conflict. This work makes an important contribution to the literature on multiple identities as, to our knowledge, it is the first to investigate whether the types of identities matter for the experience and management of conflict. Critically, this work is also the first to develop a scale to measure identity conflict management. Below we briefly review the literature on conflict and self-organization as it relates to multiple identities to build a case for examining the experience and management of conflict between multiple identities.

### Conflict between Multiple Identities: Experiences and Consequences

According to Baumeister et al. (1985), identity conflict arises when more than one identity is elicited in a given situation and these identities dictate different commitments, or sets of norms, values, motives and goals for the individual (see also Settles, 2004; Brook et al., 2008; Hirsh and Kang, 2016). Under these circumstances, individuals must contend with different ways of defining or expressing themselves, a decision that may be further complicated by the awareness that choosing one identity can come at the expense of another identity. For instance, identity conflict can occur when an individual adopts a new identity that conflicts with an old identity such as a female student in the sciences (e.g., Settles, 2004). Here prevailing norms that science is associated with men may dictate that a scientist identity contradicts a female identity (but cf. Goclowska and Crisp, 2014). Competing demands and expectations associated with two identities such as being a student and an athlete or being a parent and a professional can also promote conflict (e.g., Settles et al., 2002; Hodges and Park, 2013). Here, individuals may find it challenging to shift between two of their existing identities, leading to difficulties in the performance of one or both of these identities. Specific contexts can also cue multiple identities and highlight conflict. For instance, two existing and incompatible identities might be elicited for a lesbian-Christian attending a Sunday church sermon that condemns homosexuality; see Mahaffy, 1996; Borgman, 2009). What is striking about these examples is both the diversity of the identities that can be involved in conflict and the mundane nature of the catalysts of conflicts – Whether roles, relationships, social categories, or a combination, more than one identity can potentially be brought to mind at different times and in different contexts, potentially promoting difficulties for individuals in everyday life.

Despite having a sense of what conflict is, and when and where conflict might arise, less is known about how conflict is experienced and what it is about the identities involved that might constrain the experience of conflict. We believe that insight into these issues can be gained by considering who experiences conflict. The classic example is found in Benet- Martínez’s work on biculturals, individuals who straddle at least two different cultures (e.g., Asian-Americans; Benet-Martínez et al., 2002; Benet-Martínez and Haritatos, 2005). Here, individuals can differ in their bicultural identity integration (BII), the extent to which their two cultural identities are associated with *cultural distance*, whether cultures are (in)compatible with each other, and *cultural conflict*, whether cultures are (not) in tension with each other. High BII is associated with seeing cultural identities as compatible whereas low BII is associated with seeing cultural identities as conflictual. In addition to highlighting that some individuals may be more or less likely to experience conflict, the perceived discrepancy between identities is an important aspect of how (and whether) conflict is experienced, ultimately influencing individuals’ scores on BII. Indeed Cheng and Lee (2013) have demonstrated that the measure of BII can shift, increasing after recalling positive bicultural experiences and decreasing after recalling negative bi-cultural experiences. This finding is important because it suggests that individuals’ experience of their multiple identities can shape their evaluations of the perceived discrepancy between these identities.

This is not to say that perceiving a discrepancy between multiple identities will always lead to conflict. We know that identities derived from roles, relationships, and social groups are perceived as distinct from each other and differ on key dimensions such as whether identities are ascribed vs. achieved (i.e., automatic rather than chosen group memberships) and/or central vs. peripheral (i.e., core and important rather than marginal and unimportant; Deaux et al., 1995; see also McConnell and Strain, 2007; McConnell, 2011). In fact these differences between the types of identities may shape perceptions of their importance, valence, and flexibility – characteristics that might influence whether perceived discrepancies between multiple identities promote conflict or not. Indirect evidence that characteristics matter comes from several sources: Settles (2004) demonstrated that the importance of multiple identities moderated the magnitude of the conflict experienced. Brook et al. (2008) found that negative self-discrepant emotions mediated the relationship between conflicting multiple identities and lower psychological well-being. And Rabinovich and Morton (2016) examined individuals’ perceptions of self-flexibility as related to the experience of conflict between multiple roles. Here when the self was perceived as inflexible (i.e., as stable and fixed) experiencing conflict between multiple roles was seen as a problem. If we extend this idea about flexibility to the elements of the self rather than the overall self-concept, it is equally plausible that the perceived flexibility of identities themselves might influence the experience of conflict. Overall then, there is some suggestion that the types of identities, and their associated characteristics, might influence the experience of conflict. However, what individuals do to manage the conflict experienced between their multiple identities, and whether the types of identities and/or their characteristics are related to conflict management is less clear. To begin to address these questions we draw from existing theoretical models and empirical research to propose specific identity conflict management strategies.

#### Strategies for Managing Conflict between Multiple Identities

Identity theorists in psychological, sociological, and cultural traditions have proposed various ways that individuals might organize their multiple identities within the self or manage these identities at times of conflict and transition. For instance, in their model of *social identity complexity*, Roccas and Brewer (2002) contend that social identities differ in their degree of overlap in one of four ways: *Intersection*, where a compound identity (e.g., female-scientist) is used to define one’s ingroup, *dominance*, where one identity (e.g., scientist) serves as the superordinate category that defines the individual, *compartmentalized*, where each social identity is completely isolated from the others and only the relevant identity is elicited in a specific context (e.g., one’s scientist identity would only be activated at work but not at home), and *merged*, where both social identities would be used concurrently to define the individual in a given context (e.g., identifying both with women and scientists). In a related vein, Amiot et al. (2007, 2015) consider how pre-existing and new identities are organized within the self. In addition to categorizing themselves in terms of the new group, individuals engage in *compartmentalization*, where they keep their new and existing group memberships separate, and *integration* where elements of the new and existing groups are brought together. Similarly, Goclowska and Crisp (2014) proposed that when faced with a new identity that is similar to an existing identity, there is no change. However, when a new identity differs from an existing identity, individuals may *alternate* between these identities, *integrate* aspects of both identities into their self, or *include* the other identity into their self. As we can see integration, alternation, and compartmentalization feature as consistent ways that individuals structure multiple identities within the self.

Proposed strategies for managing conflicting multiple identities are similar to those advanced to explain how multiple identities are organized with the self. Baumeister et al. (1985) suggested that individuals might choose one identity over the other, attempt to compartmentalize their identities, separating them fully such that conflict is not possible, or try to make a compromise between their identities such that one identity is temporarily allowed to take prominence over the other, to manage the intrapsychic discomfort experienced when faced with conflicting multiple identities. Burke (2003) also identified three ways in which identities could be managed when in conflict: Individuals might choose to withdraw from one of their identities by selecting the one identity deemed more important to themselves, compromise by avoiding the situations that led to conflict between their multiple identities, or try to balance or change the meanings of both identities so that their identities approached each other. More recently Hirsh and Kang (2016) proposed that individuals could suppress the problematic identity, enhance the dominant identity, avoid situations that elicit both identities, or engage in integration as ways to manage conflict between multiple identities. Taken together these models suggest that individuals use similar strategies when trying to make sense of who they are, integrating new identities with existing identities, and in order to reduce the intrapsychic discomfort that can arise when multiple identities are elicited and conflict with each other (Baumeister et al., 1985; Roccas and Brewer, 2002; Burke, 2003; Amiot et al., 2007, 2015; Goclowska and Crisp, 2014; Hirsh and Kang, 2016).

Synthesizing the foregoing literature we propose that individuals manage conflicting multiple identities in at least three ways: *Reconciliation*, where individuals try to balance their multiple identities by integrating aspects of both identities, *Realignment*, where individuals choose one identity over another identity by selecting one identity to enact, or by focusing on one identity more than the other, and *Retreat*, where individuals avoid both of their conflicting identities, effectively compartmentalizing them. In this case, individuals may withdraw from situations where both of their identities are elicited or they may ignore both of their identities as they are incompatible with each other. These proposed identity conflict management strategies are not exhaustive, rather they represent a starting point in light of the preceding literature. Importantly, these strategies are not conceptualized as individual differences. Instead we contend that they may be used alone or in different combinations to manage conflicting multiple identities.

Although the strategies detailed above remain largely untested in relation to the experience of identity conflict, similar strategies have been examined with respect to acculturation, where individuals attempt to make sense of new and existing cultural information in order to adapt to a new context (Berry, 1997, 2005). Here individuals may engage in *assimilation*, where they choose the new culture over their existing culture, *separation*, where the existing culture is maintained while avoiding the new culture, *integration* where the new culture is adopted and the existing culture maintained, and *marginalization*, where individuals dis-identify with both their existing and new cultures. The similarities between the proposed strategies and acculturation strategies are easy to identify, with realignment reflecting aspects of assimilation, retreat reflecting aspects of marginalization and separation, and reconciliation reflecting aspects of integration. Importantly, Berry has found that integration is associated with positive adaptation to the environment whereas marginalization is associated with negative adaptation (assimilation and separation are intermediate), and argues that integration is associated with protective factors (i.e., societal and social support, flexibility in personality) relative to the other strategies, thereby encouraging its use (Berry et al., 2006; Sam and Berry, 2010). This finding suggests that all individuals might try some form of reconciliation to manage conflicting multiple identities given its benefits.

## Research Overview

The aims of the present research were threefold. First, we were interested in understanding the experience of conflict. This issue was approached in two ways: We know that conflict can occur between multiple identities, and arise for a variety of reasons, yet we do not have a sense of the types of multiple identity conflicts that typically occur. As such, we considered the nature and frequency of conflicts between multiple identities. We were also interested in understanding the experience of conflict between different types of multiple identities (e.g., pairs of roles, pairs of relationships). Here too, we have some idea that identity conflict is uncomfortable and associated with specific consequences but we do not know whether these experiences are similar across different types of identities. Second, we were interested in understanding how individuals manage conflicting multiple identities. Although existing models provide some indication of relevant identity conflict management strategies, to our knowledge, there has been little empirical research to address whether these strategies reflect the actual things that people do to manage identity conflict, or whether different types of multiple identities influence the conflict management strategies used. Third, we were interested in understanding the characteristics of multiple identities that might be related to the experience and/or management of conflict. Given that roles, relationships, and social groups differ from each other on dimensions that might be related to flexibility, importance, and valence, these characteristics might be associated with the experience and/or management of conflict. We examined these issues across three studies.

Study One provided a *descriptive account* of the frequency, experience and management of conflicts between multiple identities and was used to establish our initial hypothesis. Here participants described a current or recent identity conflict, rated its experience (i.e., magnitude, stress, and growth), and described the way(s) they managed this conflict. Studies 2 and 3 adopted an *experimental approach* to examine whether the types of multiple identities might influence the experience and management of identity conflict. In these studies participants were randomly assigned one of three conditions where they were asked to describe a conflict between two specific roles (i.e., student-employee), relationships (i.e., family-friends), or social categories (i.e., ethnicity-nationality) before rating their experience of conflict (i.e., magnitude, stress, and growth). We developed a measure of conflict management (Study 2) and examined whether types of identities in conflict influenced the use of these conflict management strategies (Studies 2 and 3). We also examined whether the characteristics of role, relational, and social identities in general (i.e., flexibility, importance, and valence) were associated with the experience and management of conflict (Study 3). Given the lack of prior empirical work on these specific relationships we developed and built on our hypotheses in stages in response to our findings. Hypotheses are presented where made below.

## Study 1: Understanding the Frequency, Experience and Management of Identity Conflict

The aim of Study 1 was to gain insight into the frequency, experience, and management of different types of identity conflict. Given the exploratory nature of these questions, specific hypotheses were not made.

### Method

#### Participants, Procedure, and Measures

Participants were 116 undergraduate psychology students (Age: *M* = 20.70; *SD* = 5.43; Gender: Female: *n* = 101; Male: *n* = 15; Race/Ethnicity: White: *n* = 48; East Asian: *n* = 22; South Asian: *n* = 14; Black: *n* = 7; Jewish: *n* = 6; Middle-Eastern: *n* = 6; South East Asian: *n* = 5; Multi-Racial: *n* = 4; Hispanic: *n* = 3) recruited from a large, suburban university in Canada for an online study on “understanding identity.” Ethical approval was obtained from the institutional research ethics committee. After completing informed consent, participants read definitions of identity, conflict, and identity conflict (see Appendix A) before selecting which of a pair of identities from a randomized list had created their most troubling conflict. The list included 11 pairs of identities derived from social psychological, developmental, and sociological literatures (e.g., ethnic-national identities, teenager-adult identities, student-employee identities; family-friend identities), an “other” option where participants could list any other pair of identities that resulted in conflict for them, and a “no conflict” option to indicate that an identity conflict had never been experienced. Eighty-nine participants (77%) reported experiencing an identity conflict. Participants who reported experiencing conflict completed open-ended questions where they described what the conflict entailed. Participants reporting an on-going conflict then described what they did to manage their experience of conflict. Participants who reported a past conflict described what they had done to resolve their conflict^1^. Responses were content coded by three independent coders (SC, JJ, and MM). Consistency of coding was computed between each pair of coders using Cohen’s Kappa where the percentage of agreement between pairs of coders ranged from 0.51 to 0.75. According to Landis and Koch (1977), this indicates moderate to substantial agreement between coders. Discrepancies were resolved by a final decision made by the first author (JJ).

Participants then rated their experience of conflict on three aspects: magnitude, stress, and growth. The measure of *magnitude*, the amount of conflict experience in general, was adapted from an existing measure of bi-cultural identity integration (BII: Benet-Martínez and Haritatos, 2005; Cheng and Lee, 2013). Items were re-worded to reflect identities in general (e.g., I feel conflicted between these two identities). The five item scale was unreliable (Cronbach’s α = 0.63) and suggested improved reliability with the removal of one item: I feel as though these two identities are combined. This left us with a 4-item scale (Cronbach’s α = 0.71; *M* = 5.13, *SD* = 1.08). Participants also rated four items to assess *stress* (i.e., how severe, difficult, challenging, troubling is/was this conflict; α = 0.91; *M* = 5.07, *SD* = 1.32), and three items to assess *growth* (i.e., how much have you learned, changed, and grown from experiencing this conflict; α = 0.83; *M* = 5.16, *SD* = 1.22). All ratings used scales from 1 *(Strongly Disagree)* to 7 *(Strongly Agree)* with higher scores indicating conflicts of greater magnitude, stress, and growth. Participants also completed demographic information (e.g., age, gender, and ethnicity) before receiving an online debriefing and our thanks.

### Results

Descriptive statistics (i.e., frequencies, means and standard deviations, correlations) were used to assess the selections, open-ended responses, and ratings provided by participants. Frequencies may be greater than a count of 89 where participants gave multiple responses.

#### Descriptions of Conflict

##### Types of identity conflict

The most frequently selected conflicts pertained to role identities (e.g., student-employee, student-athlete, employee-athlete; *n* = 37), transitional identities (i.e., teenager-adult; *n* = 20) and relational identities (e.g., couple-family, friend-family, couple-friend; *n* = 17). Social identities did not figure prominently, with bicultural identity conflicts accounting for less than 6% of the conflicts experienced (i.e., ethnicity-nationality: *n* = 5), and only one report of another type of social identity conflict (i.e., sexuality-religion). Mixed conflicts, combinations of different types of identities such as relational-social (i.e., friend-racial/ethnic; couple-racial/ethnic; *n* = 5) and role-social (i.e., gender-employee; *n* = 2), were also relatively low in frequency. Two participants indicated that they had experienced another type of identity conflict. These included a role-relational conflict (i.e., student-friend) and what can be characterized as an existential conflict (i.e., religion and life itself).

##### Reasons for identity conflict

In describing what the conflict entailed or why it occurred, 69% of participants who experienced conflict described it as comprising one issue. Thirty-one percent of participants described their conflict as comprising two or more issues. Overall, these issues took the form of time management concerns (*n* = 28), trying to balance between two identities (*n* = 16), managing others’ expectations or beliefs (*n* = 16), disapproval of one’s relationships (e.g., romantic relationships, friendships) by others (*n* = 16), transitional concerns (e.g., different life stages; *n* = 12), fighting with (or taking on) responsibilities (*n* = 11), managing personal expectations or beliefs (*n* = 6), financial concerns (*n* = 3), or other concerns (e.g., being treated in a disrespectful way by co-workers, realization that friends are changing, trying to figure out who one is; *n* = 4).

##### Managing identity conflict

In describing what was done when conflict was experienced, 79% of participants who experienced conflict indicated that one response was elicited. The remaining twenty-one percent of participants who experienced conflict reported that two or more responses were elicited. Most often, participants tried to balance their identities (*n* = 20). Participants also report attempting to ignore or avoid the conflict (*n* = 12), engaging in other positive strategies (e.g., prayer, writing, relaxing; *n* = 12), focusing on (or choosing) one identity over the other (*n* = 10), breaking-down or becoming emotional (*n* = 9), rationalizing the conflict (*n* = 8), doing nothing (*n* = 8), accepting the conflict (i.e., ‘just deal’ or ‘move on’; *n* = 7), or discussing the conflict with others (*n* = 6). Participants also reported engaging in other strategies (e.g., confronting others, adapting, becoming pensive, lying; *n* = 14) in an effort to manage identity conflict.

##### Resolving identity conflict

Twenty-one of the 89 participants who experienced conflict described this conflict as having been resolved. Thirteen of these participants reported engaging in one strategy, whereas eight participants reported engaging in two or more strategies to resolve this conflict. These responses differed from how participants reported managing their conflicts with the most common way to resolve conflict involving personal change. Individuals reported changing their attitudes or perspective (*n* = 7) or changing their lifestyle (*n* = 7) in order to resolve the conflict. Participants also reported re-evaluating the situation (*n* = 4), discussing the conflict (*n* = 4), introspection (*n* = 2), suppressing one identity (*n* = 1), or other methods (i.e., stopped trying to prove things to others; *n* = 1) as things they had done to resolve the conflict.

#### Experience of Conflict

For participants currently experiencing conflict, the magnitude, stress and growth associated with their identity conflict were rated as moderately high. Furthermore, Pearson’s correlation indicated that the magnitude of the conflict was positively related to stress from conflict and showed a marginal positive relationship with growth from the conflict (see top diagonal, **Table 1**). For participants who had resolved their conflict, the magnitude and perceived stress associated with the conflict were rated as moderate and the growth associated with their conflict was rated as moderately high. There were no relationships between the magnitude, stress, and growth associated with conflict for these individuals (see bottom diagonal, **Table 1**; also see **Table 1** for means and standard deviations).

**Table 1:** Means, standard deviations and correlations for the experience of identity conflict (current conflict – top diagonal, *n* = 68; resolved conflict – bottom diagonal, *n* = 21; Study 1).

	Current	Resolved			
					
	*M (SD)*	*M (SD)*	1	2	3
(1) Magnitude	5.32 *(1.04)*	4.49 *(0.98)*		0.54^∗∗∗^	0.24^I^
(2) Stress	5.21 *(1.31)*	4.63 *(1.29)*	0.24		0.03
(3) Growth	5.07 *(1.27)*	5.46 *(0.99)*	-0.08	0.08	


*^I^*p* < 0.07, ^∗^*p* < 0.05; ^∗∗^*p* < 0.01, ^∗∗∗^*p* < 0.001.*

### Discussion

Study 1 provides initial insights into the frequency, experience and management of conflict between multiple identities. First, our findings indicate that conflicts between role, transitional, and relational identities were most frequently reported relative to other types of identity conflicts (i.e., mixed types, social categories). This makes sense given that roles, relationships, and life-stages reflect prominent self-aspects in the minds of young adults (Arnett, 2000; McConnell, 2011). Surprisingly, conflicts between social categories or those which included social categories were not frequently reported although research on biculturals, multi-racial identities, and women in the sciences indicates that social categories such as race, ethnicity, nationality, and gender are often implicated in the experience of conflict (e.g., Settles, 2004; Benet-Martínez and Haritatos, 2005; Shih and Sanchez, 2005; Brook et al., 2008). It could be that individuals do not spontaneously think about themselves in these terms, leading social identity conflicts to be under-reported relative to conflicts involving other types of identities. Second, in rating the experience of conflict, on-going conflicts were rated moderately high on magnitude, stress, and growth. Here, magnitude was positively related to stress and magnitude also showed a marginal positive relationship with growth. These correlations suggest that experiencing conflict may be associated with adaptation, which can contribute to well-being (Tedeschi and Calhoun, 2004). Resolved conflicts were rated as moderate in magnitude and stress and moderately high on growth, but here stress and growth were unrelated to magnitude or to each other. However, these latter findings should be interpreted with caution given the relatively small numbers of individuals who reported resolved conflicts. Third, we found qualitative evidence for the proposed conflict management strategies. In particular, individuals try to balance both identities, which maps onto the idea of *reconciliation*, they tend to ignore or avoid the identities in conflict, which maps onto the idea of *retreat*, and they report focusing on one identity, which maps onto the idea of *realignment*. However, without a measure of these conflict management strategies, it is difficult to determine their use in general, or specifically when faced with particular types of identity conflicts. Lastly, personal change was implicated in the resolution of identity conflict. Although personal change as a strategy was not anticipated, its mention exclusively among individuals who had already resolved their conflicts likely reflects the end of the conflict management process. To address questions of conflict prominence and whether there might be differences in the experience and management of conflict for different types of multiple identities, our next study took a closer look at specific identity conflicts and sought to develop and test a measure of identity conflict management.

## Study 2: Do the Types of Identities Influence the Experience and Management of Conflict?

The aim of this study was to examine whether the types of multiple identities influence the experience and management of conflict. To address the prominence of different types of identity conflict, participants were randomly assigned to one of three conditions where they were explicitly asked to describe a conflict between multiple role (i.e., student-employee), relational (i.e., family-friend), or social (i.e., ethnic-national) identities. Social identity conflict was used rather than a transitional identity conflict for two reasons: First, due to its developmental nature, transitional identity conflicts reflect permanent changes that have clear indicators of onset (e.g., age, physical changes) and expected behavior(s). Transitional conflicts may therefore differ from other types of identity conflicts in how they are experienced and managed. Second, as social identity conflicts do not appear to be spontaneously generated they were included to determine if participants might report experiencing them when explicitly asked. Bi-cultural conflicts were used to represent social identity conflicts because they reflect a real concern for many young adults in multicultural societies (see Benet-Martínez et al., 2002; Benet-Martínez and Haritatos, 2005). As in Study 1, the experience of conflict was assessed using measures of magnitude, stress, and growth. Conflict management was assessed using a new scale developed for the present study. In light of Study 1 where ratings of magnitude, stress and growth were moderate to high and all participants reported attempts to manage conflict, we expected that conflict magnitude, stress and growth would be positively related the conflict management strategies (*Hypothesis 1*). We also considered whether the types of identities might influence the experience and management of conflict. Given that people’s role, relational, and social identities cluster along different dimensions (Deaux et al., 1995), it is plausible that the types of identities might also be associated different conflict experiences and management strategies. However, as there is no prior theoretical work or empirical evidence to suggest the direction of these effects specific hypotheses were not made about how these variables might be related to each other.

### Method

#### Participants, Procedure, and Measures

Participants were 366 undergraduates recruited for an online study on ‘understanding identity’ from a large suburban university in Canada (Age: *M* = 20.75; *SD* = 3.50; Gender: 64% Female; Race/Ethnicity: White: *n* = 151; South Asian: *n* = 61; East Asian: *n* = 66; Multi-Racial: *n* = 20; Middle-Eastern: *n* = 12; Black: *n* = 24; Hispanic: *n* = 12; Native-Canadian: *n* = 1; Missing: *n* = 3). The study employed a 3 Type of Identities (Role, Relational, and Social) single factor between-subjects design. Participants received either course credit or $5 Canadian dollars for completion of this study. Ethical approval was obtained through the institutional research ethics committee.

After completing informed consent, participants provided details about different aspects of themselves (i.e., age, gender, student, employee status, friend, family, race, ethnicity, religion, and relationship status) and rated the importance of these aspects (e.g., *Being a student* is an important part of my identity). Participants were then randomly assigned to describe a conflict between role (i.e., *student-employee*), relational (i.e., *friend-family*) or social (i.e., *ethnicity-nationality*) identities. If participants indicated that they had not experienced the identity conflict to which they had been assigned, they had the option of selecting an identity conflict from a list of conflicts, with one of these options allowing them to specify any other type of identity conflict or that they had never experienced conflict. These options were the same as those used in Study 1. One-hundred and fifty-one participants indicated that they experienced the conflict to which they had been assigned (role: *n* = 49; relational: *n* = 53; social: *n* = 49). Forty-six participants (12.6%) indicated that they had never experienced an identity conflict and were directed to the end of the questionnaire. One-hundred and sixty-nine participants indicated they had experienced a conflict other than the one to which they had been assigned. Of these participants, fifty-five subsequently selected one of the target role, relational, or social conflicts (student-employee: *n* = 26; friend-family: *n* = 18; ethnicity-nationality: *n* = 11). The remaining participants selected another type of conflict: relationships (couple-family: *n* = 16; friend-couple: *n* = 12), social (multi-racial: *n* = 2), transitional (teenager-adult: *n* = 37), mixed types of conflicts (i.e., role-relational – student-couple: *n* = 22, student-friend: *n* = 1; social-role – gender-student: *n* = 2; social-relational – couple-race: *n* = 8; friend-race: *n* = 4; couple-religion: *n* = 1), or wrote in their own conflicts (*n* = 10; e.g., the self and one identity; tri-identity conflicts).

Findings are presented for the 205 participants who reported experiencing the target conflicts between roles (i.e., student-employee), relationships (i.e., friend-family), and social categories (i.e., culture-nationality). Fifty-seven of these conflicts were described as on-going whereas 148 were described as recent or past. One person who had been assigned to the culture-nationality identity condition did not complete the questionnaire and was omitted from further analyses. After describing their conflict, participants rated its experience on three dimensions from 1 *Strongly Disagree* to 5 *Strongly Agree*: *magnitude* (see *Study 1*: four items; Cronbach’s α = 0.81), *stress* (seven items; i.e., how severe/difficult/challenging/troubling/bothersome/demanding/stressful is this conflict; Cronbach’s α = 0.94), and *growth* (see *Study 1*: three items; Cronbach’s α = 0.84) as well as rating its management.

To assess conflict management a new measure was developed on the basis of the previous literature (e.g., Baumeister et al., 1985; Roccas and Brewer, 2002; Burke, 2003), participants’ responses from Study 1, and in consultation with other identity researchers. To ensure representativeness across different types of conflicts, responses from all participants who reported an identity conflict (i.e., *n* = 319) were used in the factor analysis of an initial set of 32 items (principal axis, direct oblimin rotation, pattern matrix interpreted using a 0.50 loading cut-off). Fifteen items were distributed across the first five factors: *Retreat*, the avoidance and compartmentalization of conflicting identities (16.13% of the variance; four items; *Cronbach’s*α = 0.81), *Reconciliation*, trying to balance between, or integrate, conflicting identities (12.95% of the variance; four items; *Cronbach’s*α = 0.73), *Realignment*, choosing one identity over the other 11.62% of the variance; three items; *Cronbach’s*α = 0.74), *Reflection*, using the identity accommodates others or the situation (12.54% of the variance; two items; *r* = 0.55, *p <* 0.001), and *Relinquishment*, giving up one or both identities (4.96% of the variance; two items; *r* = 0.28, *p <* 0.001; see Appendix B for items and factor loadings)^2^.

### Results

Descriptive statistics (i.e., means and standard deviations, correlations) are reported in **Table 2**.

**Table 2:** Means, standard deviations and correlations for the experience and management of identity conflict (Study 2).

	*M (SD)*	*2*	*3*	*4*	*5*	*6*	*7*	*8*
(1) Magnitude	2.84 *(0.85)*	0.56^∗∗∗^	0.32^∗∗∗^	0.43^∗∗∗^	0.14^I^	0.16^∗^	0.36^∗∗∗^	-0.06
(2) Stress	3.01 *(0.86)*		0.46^∗∗∗^	0.41^∗∗∗^	0.13^I^	0.19^∗∗^	0.18^∗∗^	0.09
(3) Growth	3.43 *(0.86)*			0.15^∗^	0.16^∗^	0.06	0.20^∗∗^	0.04
(4) Retreat	2.98 *(0.80)*				0.01	0.41^∗∗∗^	0.37^∗∗∗^	0.03
(5) Reconciliation	3.53 *(0.67)*					-0.12	0.19^∗∗^	0.08
(6) Realignment	3.16 *(0.86)*						0.20^∗∗^	-0.04
(7) Reflection	3.27 *(0.92)*							0.02
(8) Relinquishment	3.36 *(0.78)*							


*^I^*p* < 0.07, ^∗^*p* < 0.05, ^∗∗^*p* < 0.01, ^∗∗∗^*p* < 0.001. Controlling for conflict occurrence, conflict choice, and gender.*

#### Relationships between the Experience and Management of Identity Conflicts

Partial correlations were used to examine the relationships between the experience and management of conflict controlling for conflict occurrence (on-going, recent/past), conflict choice (assigned, selected) and gender (male, female; see **Table 2**). In terms of the experience of conflict, magnitude, stress, and growth were all positively related to each other. In terms of the conflict management strategies, retreat was positively related to realignment and reflection. Reflection was positively related to reconciliation and realignment. Relinquishment was not related to any of the other management strategies. In support of *Hypothesis 1*, the experience of conflict was positively related to the management of conflict: As the magnitude of conflict increased, the use of retreat, realignment, and reflection strategies increased and reconciliation marginally increased. Similarly, as stress increased, the use of retreat, realignment, reflection strategies increased and reconciliation marginally increased. Finally, as growth increased, the use of the retreat, reconciliation, and reflection strategies increased.

#### Experiencing Conflict between Multiple Identities

A 3 Type of Identities (Role, Relational, and Social) single-factor between-subjects ANCOVA with conflict occurrence, conflict choice and gender as covariates was used to test whether the experience of conflict differed as a function of the identities in conflict. All pairwise comparisons were conducted with Least Significant Difference (LSD).

##### Conflict magnitude

The magnitude of conflict did not differ as a function of the types of identities in conflict, *F*(2,199) = 1.93, *p* = 0.148. Participants experienced conflicts of similar magnitude regardless of the types of identities in conflict (Role: *M* = 2.84, *SE* = 0.098, Relational: *M* = 2.71, *SE* = 0.099, Social: *M* = 3.00, *SE* = 0.108; all *p*s > 0.051).

##### Perceived stress

Stress did not differ as a function of the types of identities in conflict, *F*(2,199) = 1.02, *p* = 0.364. Participants experienced similar levels of stress regardless of the types of identities in conflict (Role: *M* = 3.11, *SE* = 0.101, Relational: *M* = 2.90, *SE* = 0.103, Social: *M* = 3.00, *SE* = 0.112; all *p*s > 0.156).

##### Perceived growth

Growth did not differ as a function of the types of identities in conflict, *F*(2,199) = 2.46, *p* = 0.09. Although the overall effect was not significant, pairwise comparisons suggested that role conflicts were associated with less growth (*M* = 3.26, *SE* = 0.100) than were social conflicts (*M* = 3.57, *SE* = 0.110; *p* = 0.038). Relational conflicts (*M* = 3.50, *SE* = 0.101) did not differ in magnitude relative to role or social conflicts (both *p*s > 0.096).

#### Managing Conflict between Multiple Identities

A 3 Type of Identities (Role, Relational, and Social) × 4 Conflict Management (Reconciliation, Realignment, Retreat, and Reflection) mixed ANCOVA, with type of identities as the between factor, management strategy as the within factor, and conflict occurrence, conflict choice, and gender as covariates was used to test whether the management of conflict differed as a function of the identities in conflict. Relinquishment was not included as it was unrelated to the other conflict management strategies. All pairwise comparisons were conducted with LSD.

Although there was no main effect for type of identities, *F*(2,199) = 1.96, *p* = 0.14, a main effect for management strategy emerged, *F*(3,597) = 3.12, *p* = 0.025, $\eta_{p}^{2}$ = 0.015: The use of reflection did not differ from the use of realignment (*p* = 0.134). The use of all other management strategies were significantly different from each other. Overall, participants used reconciliation more relative to all other management strategies (all *p*s < 0.006) and retreat less relative to all other management strategies (all *p*s < 0.002). This effect was qualified by the significant Type of Identities by Management Strategy interaction, *F*(6,597) = 2.33, *p* = 0.031, $\eta_{p}^{2}$ = 0.023. To explore this interaction, four single factor between-subjects ANCOVAs, using each management strategy as a dependent variable, Type of Identities as the between subjects factor, and conflict occurrence, conflict choice, and gender as covariates, were conducted.

##### Retreat

There was no main effect for the Type of Identities on retreat, *F*(2,199) = 1.77, *p* = 0.17, $\eta_{p}^{2}$ = 0.017. Participants were equally likely to engage in this strategy, regardless of the types of identities in conflict (Role: *M* = 3.12, *SE* = 0.095, Relational: *M* = 2.90, *SE* = 0.096, Social: *M* = 2.89, *SE* = 0.105; all *p*s > 0.102).

##### Reconciliation

There was no main effect for the Type of Identities on reconciliation, *F*(2,199) = 0.238, *p* = 0.788, $\eta_{p}^{2}$ = 0.002. Participants were equally likely to engage in this strategy, regardless of the types of identities in conflict (Role: *M* = 3.52, *SE* = 0.079, Relational: *M* = 3.50, *SE* = 0.080, Social: *M* = 3.58, *SE* = 0.087; all *p*s > 0.498).

##### Realignment

A main effect for Type of Identities was found on realignment, *F*(2,199) = 5.79, *p* = 0.004, $\eta_{p}^{2}$ = 0.055: Participants who described conflicts between role identities reported more realignment (*M* = 3.42, *SE* = 0.099), relative to participants who described conflicts between relational identities (*M* = 2.94, *SE* = 0.101; *p* = 0.001), and social identities (*M* = 3.10, *SE* = 0.110; *p* = 0.034). There were no differences in the use of realignment for conflicts between relational and social identities (*p* = 0.274).

##### Reflection

There was no main effect for the Type of Identities on reflection, *F*(2,199) = 0.549, *p* = 0.578, $\eta_{p}^{2}$ = 0.005. Participants were equally likely to engage in this strategy, regardless of the types of identities in conflict (Role: *M* = 3.20, *SE* = 0.11, Relational: *M* = 3.24, *SE* = 0.111, Social: *M* = 3.37, *SE* = 0.121; all *p*s > 0.313).

We also looked at relinquishment as a separate dependent variable. Here a main effect for Type of Identities was found, *F*(2,199) = 5.47, *p* = 0.005, $\eta_{p}^{2}$ = 0.052: Participants who described conflicts between social identities reported less relinquishment (*M* = 3.10, *SE* = 0.10) relative to participants who described conflicts between role identities (*M* = 3.39, *SE* = 0.09; *p* = 0.033) or relational identities (*M* = 3.54, *SE* = 0.092; *p* = 0.001). There were no differences in the use of relinquishment for conflicts between role and relational identities (*p* = 0.255).

### Discussion

Study 2 provides important information about the experience and management of conflicts between different types of multiple identities. First, in considering our new measure of conflict management, we found that reconciliation was used most, and retreat was used least, relative to all other strategies. This suggests that people generally try to balance or integrate their multiple identities to manage identity conflict. Although relinquishment also emerged as a factor, as it was unrelated to the other management strategies it could be that relinquishment reflects a post-conflict outcome rather than a way of (actively) managing conflict. Some evidence to support this idea can be seen in additional analyses looking at individuals who reported that their conflicts were in the past (i.e., 7+ months ago). Here relinquishment was positively related to stress and growth and showed a marginal positive relationship with reconciliation. These relationships were not found for individuals who reported on-going or recent (i.e., 6 or less months) conflicts. Second, and in support of *Hypothesis 1*, the experience of conflict was positively related to its management: Although conflict magnitude, stress and growth were all positively related to retreat, realignment, and reflection, only growth was positively related to reconciliation. It could be that when contending with identity conflicts, individuals might gravitate toward strategies that enable them to avoid the conflicting identities or focus on one of these identities over the other. Yet when individuals have had some time to *live with* their conflicts, as might be suggested by experiencing growth, the extent to which they balance their conflicting identities might also increase. Third, the experience of conflict did not differ as a function of the types of identities in conflict. However, we did find evidence for the differential use of management strategies as a function of the types of identities in conflict. Here role identities were associated with greater use of realignment relative to relational or social identity conflicts. Reconciliation, reflection and retreat were not differentially used. Taken together, we have preliminary evidence that the types of identities matter for the management, but not necessarily for the experience, of identity conflict, with multiple roles lending themselves to more realignment relative to relationships or social categories. However, we do not know what it is about roles that might allow for the use of realignment relative to other types of identities. In addition to establishing the stability of our measure of identity conflict management, our subsequent study examined whether the perceived characteristics of individuals’ identities in general might be associated with differences in the experience and management of conflict.

## Study 3: Identity Characteristics and the Experience and Management of Conflict

The aim of Study 3 was twofold. In addition to examining the experience and management of conflict in another sample, we first sought to examine the stability of the measure of conflict management. Finding the same factors and similar reliabilities would go some way to confirming that these strategies reflect a meaningful measurement in the context of identity conflicts. Second, we sought to examine whether the characteristics of role, relational and social identities in general might be associated with the experience and management of conflict. We already know that the sources of identities (i.e., social categories, social groups, relationships, roles) are perceived as distinct from each other and differ on key dimensions including flexibility and importance (Deaux et al., 1995; McConnell and Strain, 2007; McConnell, 2011). And recent work has shown that the perceived importance and valence associated with multiple identities matters for the experience of conflict (Settles, 2004; Brook et al., 2008). When considered along with our initial evidence that role identities differ from relational and social identities in the use of realignment it could be that the general perceptions of different types of identities shape the specific strategies used in management of conflict. However, at least based on our initial findings, there is little evidence to suggest that these perceptions might influence the experience of conflict.

To examine these relationships, we had participants rate the perceived flexibility, valence, and importance of their identities in general before being randomly assigned to one of three conditions where they were explicitly asked to describe a conflict between multiple role, relational, or social identities, before rating their experience of conflict (magnitude, stress, and growth) and their use of conflict management strategies (retreat, reconciliation, realignment, and reflection). Given the findings from Study 2 we expected a positive relationship between the experience and management of conflict, with conflict magnitude, stress, and growth showing positive relationships with each of the conflict management strategies *(Hypothesis 1)*. Given the findings from Study 2 we also expected that the types of identities in conflict would influence the management of conflict (i.e., reconciliation, realignment, retreat, and reflection) but not the experience of conflict (i.e., magnitude, stress, and growth). In particular, we expected that conflicts between multiple role identities would be associated with more realignment relative to conflicts between multiple relational or social identities (*Hypothesis 2*).

In extension of Study 2, we considered whether the characteristics of identities in general were associated with the experience and management of conflict. Given the findings from Study 2 and previous research which suggested that identities differ on dimensions related to flexibility, importance, and valence, and that these characteristics are related to the experience of conflict (e.g., Deaux et al., 1995; Settles, 2004; Brook et al., 2008; McConnell, 2011), we expected that the general perception that one’s identities are more flexible and less important would be associated with conflicts of less magnitude, stress, and growth (*Hypotheses 3a*) and the use of realignment and retreat (*Hypothesis 3b*) particularly when these characteristics were associated with roles rather than relationships or social categories.

### Method

#### Participants, Procedure, and Measures

Participants were 300 adults recruited for an online study on ‘understanding identity’ via Prolific Academic, a crowd-sourcing participant recruitment website. Seven participants identified as duplicates (the same IP address and location data for more than one entry) and three participants who selected but did not write about a conflict were excluded leaving us with a sample of 290 (Age: *M* = 24.76; *SD* = 6.41; Gender: 46% Female, 2% Agender; Race/Ethnicity: White: *n* = 203; East Asian: *n* = 27; Black: *n* = 14; Hispanic: *n* = 10; Multi-Racial: *n* = 9; South Asian: *n* = 5; Middle-Eastern: *n* = 1; Missing: *n* = 21; Nationality: American: *n* = 144, British: *n* = 88, Canadian: *n* = 18; Other: *n* = 40; Student: Yes: *n* = 219, No: *n* = 71; Employed: Yes: *n* = 170, No: *n* = 120). The study employed a 3 Type of Identities (Role, Relational, and Social) single factor between-subjects design. Participants received two British pounds sterling for completion of this study. Ethical approval was obtained through the institutional research ethics committee.

After completing informed consent, participants provided demographic information (e.g., age, gender, and ethnicity) as well as details about different aspects of themselves (i.e., student, employee, friend, family, relationship, race, ethnicity, nationality, and religion) and the perceived flexibility (e.g., My *student* identity is: Very Inflexible – Very Flexible), importance (two items each: e.g., I identify with my *family, My* family is an important part of how I see myself) and valence (e.g., My *ethnic* identity is: Very Negative – Very Positive) of these identities.^3^ Participants then read a description of an identity conflict (see Appendix A), indicated whether they had read the description (i.e., Y/N) and indicated whether, upon reading the description, “I understand the definition of identity conflict and how it might apply to me” on a scale from 1 *Strongly Disagree* to 5 *Strongly Agree* (*M* = 4.48, *SD* = 0.53). As in Study 2, participants were then randomly assigned to describe a conflict between their role (i.e., *student-employee*), relational (i.e., *friend-family*), or social (i.e., *ethnicity-nationality*) identities. If participants indicated that they had not experienced the identity conflict to which they had been assigned, they had the option of selecting an identity conflict from a list of conflicts, with one of these options allowing them to specify any other type of identity conflict or that they had never experienced conflict. One-hundred and sixty-nine participants indicated that they experienced the conflict to which they had been assigned (role: *n* = 65; relational: *n* = 73; social: *n* = 31). Ninety-nine participants indicated that they had experienced a conflict other than the one to which they had been assigned. Of these participants, 19 selected one of the main role or relational conflicts of interest (student-employee: *n* = 11; friend-family: *n* = 9). No participants selected the main social identity conflict (i.e., ethnicity-nationality). The remaining 80 participants selected another type of conflict: roles (student-recreational: *n* = 16), relationships (couple-family: *n* = 12), social (two races/ethnicities: *n* = 7; sexuality-religion: *n* = 3; religion-nationality: *n* = 1), transitional (two-life stages: *n* = 17), mixed types (social-role: gender-student: *n* = 6; social-relational: couple-race/ethnicity: *n* = 3; friend-race/ethnicity: *n* = 4), and other (*n* = 11; e.g., conflicts involving the self and one identity; conflicts between three identities). Twenty-two participants indicated that they never experienced conflict between any of their identities. Findings are presented for 188 participants who were assigned to, or subsequently selected, conflicts between roles (i.e., student-employee), relationships (i.e., friend-family), and social categories (i.e., ethnicity-nationality). Seventy-seven of these conflicts were current whereas 111 were resolved. Findings for the individuals who reported other types of identity conflicts (*n* = 80) are not reported.

After describing the conflict, participants rated their experience of conflict on three dimensions: *magnitude* (see *Study 1*: four items; Cronbach’s α = 0.82, *stress* (see *Study 2*: seven items, Cronbach’s α = 0.88), and *growth* (see *Study 1*: three items; Cronbach’s α = 0.85). To assess the management of identity conflict, the 13 correlated items from Study 2 were used. Factor analysis (principal axis, direct oblimin rotation, pattern matrix interpreted using a 0.40 loading cut-off) suggested a four-factor solution: *Reconciliation* (19.84% of the variance; four items; *Cronbach’s*α = 0.77), *Realignment* (16.25% of the variance; three items; *Cronbach’s*α = 0.72), *Retreat* (16.09% of the variance; four items; *Cronbach’s*α = 0.76), and *Reflection* (12.02% of the variance; two items; *r* = 0.59, *p* < 0.001; see Appendix C for items and factor loadings). All ratings were completed on scales from 1 *Strongly Disagree* to 5 *Strongly Agree*.

For the identity characteristics, composites were created for *student importance* [two items; *r*(145) = 0.57, *p* < 0.001], *employee importance* [two items; *r*(116) = 0.75, *p* < 0.001], *family importance* [two items; *r*(188) = 0.84, *p* < 0.001], *friend importance* [two items; *r*(185) = 0.61, *p* < 0.001], *ethnic importance* [two items; *r*(186) = 0.70, *p* < 0.001], and *national importance* [two items; *r*(187) = 0.73, *p* < 0.001]. In line with the key conflicts of interest, these were then averaged to created composites for *role importance* [student, employee; *r*(86) = 0.25, *p* = 0.021], *relational importance* [family, friends; *r*(188) = 0.27, *p* < 0.001], *social importance* [ethnicity, nationality; *r*(188) = 0.52, *p* < 0.001], *role valence* [student, job; *r*(85) = 0.23, *p* = 0.033], *relational valence* [family, friends; *r*(187) = 0.22, *p* = 0.003], *social valence* [ethnicity, nationality; *r*(187) = 0.50, *p* < 0.001], *relational flexibility* [family, friends; *r*(187) = 0.19, *p* = 0.010] and *social flexibility* [ethnicity, nationality; *r*(187) = 0.35, *p* < 0.001]. As *student flexibility* and *employee flexibility* were unrelated to each other [*r*(146) = 0.014, *p* = 0.898], they were used as individual items.

### Results

#### Relationships between Characteristics and the Experience and Management of Conflict

Partial correlations were used to examine the relationships between identity characteristics, and the experience and management of conflict controlling for conflict occurrence, conflict choice, and gender (see **Table 3** *for means and standard deviations; see* **Table 4** *for correlations*). As in Study 1, conflict magnitude, stress, and growth were all positively related to each other. In support of *Hypothesis 1*, and similar to the results of Study 2, the experience of conflict was positively related to the management of conflict: As the magnitude of conflict increased, the use of retreat and reconciliation strategies increased. As stress increased, the use of retreat and realignment strategies increased. However, in contrast to Study 2, growth was unrelated to the conflict management strategies.

**Table 3:** Means and standard deviations for identity characteristics, and the experience and management of identity conflict (Study 3).

*Ratings*	*n*	*M (SD)*
Student flexibility	146	3.61 *(0.90)*
Employee flexibility	116	3.03 *(0.97)*
Relational flexibility	188	3.23 *(0.81)*
Social flexibility	188	2.86 *(0.89)*
Role importance	176	3.60 *(0.86)*
Relational importance	188	3.90 *(0.76)*
Social importance	188	3.61 *(0.85)*
Role valence	176	3.79 *(0.76)*
Relational valence	188	3.94 *(0.72)*
Social valence	188	3.63 *(0.79)*
Conflict magnitude	187	3.10 *(0.91)*
Stress	188	3.44 *(0.83)*
Growth	188	3.58 *(0.92)*
Retreat	188	3.17 *(0.88)*
Reconciliation	188	3.62 *(0.79)*
Realignment	188	3.45 *(0.90)*
Reflection	188	3.37 *(0.97)*
Valid N (Listwise)	86	


**Table 4:** Correlations between identity characteristics, and the experience and management of identity conflict (Study 3).

	2	3	4	5	6	7	8	9	10	11	12	13	14	15	16	17
(1) Student flexibility	-0.00	0.19^∗^	0.21^∗^	-0.00	-0.01	0.10	0.19^∗^	0.03	0.14	0.16^I^	0.27^∗∗^	0.25^∗∗^	0.18^∗^	-0.06	0.17^∗^	0.07
(2) Employee flexibility		0.21^∗^	0.16	0.05	0.05	0.16	0.29^∗∗^	0.23	0.14	-0.02	0.06	0.07	-0.02	0.16	-0.03	0.07
(3) Relational flexibility			0.36^∗∗∗^	0.10	0.10	0.07	0.13	0.07	0.05	-0.12	-0.04	0.00	0.15^∗^	0.12	0.01	0.11
(4) Social flexibility				-0.11	-0.09	-0.02	-0.10	-0.04	0.12	0.02	0.04	0.15^∗^	0.08	0.02	-0.01	-0.03
(5) Role importance					0.36^∗∗∗^	0.23^∗∗^	0.56^∗∗∗^	0.34^∗∗∗^	0.18^∗^	0.10	0.09	0.08	0.06	0.24^∗∗^	-0.08	0.09
(6) Relational importance						-0.22^∗∗^	0.20^∗∗^	0.76^∗∗∗^	0.16^∗^	0.11	-0.04	-0.04	-0.03	0.33^∗∗∗^	-0.12	0.34^∗∗∗^
(7) Social importance							0.25^∗∗^	0.19^∗^	0.66^∗∗∗^	0.15^∗^	0.01	0.18^∗^	-0.04	0.22^∗∗^	0.03	0.05
(8) Role valence								0.29^∗∗∗^	0.18^∗^	0.13	0.24^∗∗^	0.08	0.10	0.14^I^	0.11	-0.02
(9) Relational valence									0.26^∗∗∗^	0.16^∗^	0.01	0.09	-0.01	0.32^∗∗∗^	-0.20^∗∗^	0.24^∗∗^
(10) Social valence										0.18^∗^	-0.02	0.23^∗∗^	0.02	0.12	-0.04	-0.01
(11) Magnitude											0.35^∗∗∗^	0.23^∗∗^	0.31^∗∗∗^	0.22^∗∗^	-0.06	0.13
(12) Stress												0.37^∗∗∗^	0.33^∗∗∗^	0.02	0.16^∗^	-0.03
(13) Growth													0.08	0.08	-0.01	-0.13
(14) Retreat														-0.02	0.10	0.25^∗∗∗^
(15) Reconciliation															-0.40^∗∗∗^	0.27^∗∗∗^
(16) Realignment																-0.10
(17) Reflection																


*Controlling for conflict occurrence, conflict choice, and gender. ^I^*p* < 0.07, ^∗^*p* < 0.05, ^∗∗^*p* < 0.01, ^∗∗∗^*p* < 0.001. Controlling for conflict occurrence, conflict choice, and gender.*

In partial support of *Hypothesis 3a*, identity characteristics were related to the experience of conflict. However, these relationships were in the opposite direction to our hypotheses: Student identity flexibility, a role, was *positively* related to stress and growth and marginally *positively* related to conflict magnitude. Unexpectedly, we also found that social identity flexibility was positively related to growth and that social identity importance was positively related to conflict magnitude, stress, and growth. In terms of valence, role identity valence was positively related to stress, relational identity valence was positively related to conflict magnitude, and social identity valence was positively related to conflict magnitude, stress, and growth. In partial support of *Hypothesis 3b*, student identity flexibility was positively related to retreat and realignment and relational identity flexibility was positively related to retreat. For importance, role, relational, and social identity importance were all positively related to reconciliation, with relational identity importance also showing a positive relationship with reflection. In terms of valence relational identity valence was positively related to reconciliation and reflection but negatively related to realignment. Role identity valence was marginally associated with reconciliation.

#### Experiencing Conflict between Multiple Identities

A 3 Type of Identities (Role, Relational, and Social) single-factor between-subjects ANCOVA with conflict occurrence, conflict choice, and gender as covariates was used to test whether the experience of conflict differed as a function of the identities in conflict. All pairwise comparisons were conducted with LSD.

##### Conflict magnitude

Similarly to Study 2, conflict magnitude did not differ as a function of the types of identities in conflict, *F*(2,180) = 2.55, *p* = 0.081. Although the overall effect was not significant, pairwise comparisons suggested that role conflicts were of greater magnitude (*M* = 3.25, *SE* = 0.099) than were social conflicts (*M* = 2.84, *SE* = 0.158; *p* = 0.030). Relational conflicts (*M* = 3.06, *SE* = 0.097) did not differ in magnitude relative to role or social conflicts (both *p*s > 0.177).

##### Perceived stress

Contrary to Study 2, stress differed as a function of the types of identities in conflict, *F*(2,181) = 6.95, *p* = 0.001, $\eta_{p}^{2}$ = 0.071: Role conflicts were associated with more stress (*M* = 3.70, *SE* = 0.092) relative to relational conflicts (*M* = 3.32, *SE* = 0.09; *p* = 0.004) or social conflicts (*M* = 3.14, *SE* = 0.15; *p* = 0.001). The stress associated with relational and social identity conflicts were not different from each other (*p* = 0.30).

##### Perceived growth

Similarly to Study 2, growth did not differ as a function of the types of identities in conflict, *F*(2,181) = 0.62, *p* = 0.54. Participants experienced similar levels of growth regardless of the types of identities in conflict (Role: *M* = 3.62, *SE* = 0.11, Relational: *M* = 3.48, *SE* = 0.111, Social: *M* = 3.68, *SE* = 0.172; all *p*s > 0.339).

#### Managing Conflict between Multiple Identities

A 3 Type of Identities (Role, Relational, and Social) × 3 Conflict Management (Reconciliation, Realignment, Retreat, and Reflection) mixed ANCOVA, with type of identities as the between factor, management strategy as the within factor, and conflict occurrence, conflict choice, and gender as covariates was used to test whether the management of conflict differed as a function of the identities in conflict. All pairwise comparisons were conducted with LSD.

Although the main effects for management strategies and types of identities were not statistically significant, both *F*s < 0.76, both *p*s > 0.52, in support of *Hypothesis 2*, and in line with Study 2, the Type of Identities by Management Strategy interaction was significant, *F*(6,543) = 2.18, *p* = 0.044, $\eta_{p}^{2}$ = 0.023. To explore this interaction, four single factor between-subjects ANCOVAs, using each management strategy as a dependent variable, Type of Identities as the between subjects factor, and conflict occurrence, conflict choice and gender as covariates, were conducted.

##### Retreat

In contrast to Study 2, a main effect for Type of Identities was found on retreat, *F*(2,181) = 5.28, *p* = 0.006, $\eta_{p}^{2}$ = 0.055: Participants who described conflicts between relational identities engaged in more retreat (*M* = 3.40, *SE* = 0.10), relative to participants who described conflicts between role identities (*M* = 3.07, *SE* = 0.10; *p* = 0.021), and social identities (*M* = 2.83, *SE* = 0.16; *p* = 0.003). There were no differences in the use of retreat for conflicts between role and social identities (*p* = 0.224).

##### Reconciliation

Similarly to Study 2, there was no main effect for the Type of Identities on reconciliation, *F*(2,181) = 0.40, *p* = 0.67, $\eta_{p}^{2}$ = 0.00. Participants were equally likely to engage in this strategy, regardless of the types of identities in conflict (Role: *M* = 3.59, *SE* = 0.093, Relational: *M* = 3.59, *SE* = 0.090, Social: *M* = 3.74, *SE* = 0.148; all *p*s > 0.399).

##### Realignment

In contrast to Study 2, there was no main effect for the Type of Identities on realignment, *F*(2,181) = 1.27, *p* = 0.28, $\eta_{p}^{2}$ = 0.014. Participants were equally likely to engage in this strategy, regardless of the types of identities in conflict (Role: *M* = 3.59, *SE* = 0.104, Relational: *M* = 3.40, *SE* = 0.102, Social: *M* = 3.32, *SE* = 0.166; all *p*s > 0.177).

##### Reflection

Similarly to Study 2, there was no main effect for the Type of Identities on reflection, *F*(2,181) = 0.21, *p* = 0.81, $\eta_{p}^{2}$ = 0.00. Participants were equally likely to engage in this strategy, regardless of the types of identities in conflict (Role: *M* = 3.31, *SE* = 0.114, Relational: *M* = 3.38, *SE* = 0.111, Social: *M* = 3.44, *SE* = 0.182; all *p*s > 0.545).

### Discussion

Study 3 provides evidence for the stability of our measures of conflict experience and management as well as important insights into the experience and management of conflict, and their relationships with identity characteristics. With a markedly less diverse and slightly older sample relative to Studies 1 and 2, we found similar reliabilities for the measures of conflict experience and these measures were all positively related to each other. This suggests that magnitude, stress, and growth are useful measures for capturing the experience of conflict between multiple identities. Critically, we also found the same factors and similar reliabilities for the conflict management strategies. This suggests that retreat, reconciliation, realignment, and reflection are strategies that people use to manage conflicts between multiple identities. However, the present sample differed in the distribution identity conflicts, with almost 50% fewer bi-cultural conflicts being described relative to Study 2. Unsurprisingly, with these differences in the distribution of conflicts, we found both consistent and inconsistent results for the experience and management of conflicts between different types of multiple identities^4^.

First, in support of *Hypothesis 1*, the experience of conflict was positively related to its management. Conflict magnitude was positively related to retreat and reconciliation, and stress was positively related to retreat and realignment. However, growth was unrelated to any management strategies. While these relationships still support the idea that individuals gravitate toward a strategy that enables them to choose one identity or avoid their conflicting identities, especially when they are associated with stress, it also suggests that as perceptions of the discrepancy between multiple identities increase (i.e., increases in magnitude), there are increased attempts to integrate them. Despite some different relationships relative to Study 2 (i.e., conflict magnitude was unrelated to realignment or reflection, growth was unrelated to any of the management strategies), the patterns from the present study still support the overall assertion that the experience of conflict is positively related to the management of conflict.

Second, we unexpectedly found that the experience of conflict differed as a function of the types of identities in conflict. Here, conflicts between role identities were associated with more stress relative to conflicts between relational or social identities. There were no differences in magnitude or growth by the types of identities in conflict. As this finding differed from Study 2 where no relationship was found, we considered whether characteristics of the conflict or samples might shed light on this discrepancy. Unfortunately, we could not find any reasons for why this might be the case: In Studies 2 and 3 the number of conflicts that were on-going vs. resolved was similar, and when supplementary analyses controlled for additional participant characteristics (i.e., age, race), the results remained the same.

Third, and in support of *Hypothesis 2*, the management of conflict differed as a function of the types of identities in conflict. Here it was retreat that was used more to manage relational conflicts relative to role or social identity conflicts. Reconciliation, realignment, and reflection were not differentially used depending on the types of identities in conflict. Although different to Study 2, these pattern of these findings add to, rather than challenge, our understanding of the management strategies for different types of identities in conflict. It suggests that retreat might be used more when contending with relational conflicts whereas realignment might be used more with role conflicts. This makes sense as it is likely more difficult to extricate one’s self from relationships than from roles which can explain the reliance on different management strategies for different types of identity conflicts.

Fourth, we investigated whether identity characteristics in general might be associated with the experience and management of conflict. In partial support of *Hypothesis 3a*, student identity flexibility, a role, was *positively* related to the experience of conflict and social identity flexibility was positively related to growth. Although this was opposite to the direction expected, as the flexibility of employee identity, relational identities, and social identities were generally unrelated to the majority of the conflict experience measures it lends support to the idea that perceived flexibility is a key aspect of the experience of conflict. In partial support of *Hypothesis 3b*, student identity flexibility was positively related to realignment and retreat. Relational identity flexibility was also positively related to retreat. These relationships are generally consistent with the conflict management findings of Studies 2 and 3 and lend credence to our earlier assertion that flexibility is related tied to the strategic use of conflict management strategies. We also found that more important identities and more positive identities, regardless of type, were generally associated with reconciliation, suggesting that the perceived value of identities may influence whether individuals use this strategy. Furthermore, exploratory analyses^5^ also suggested that role identities were perceived as more flexible relative to relational and social identities, and that relational identities were perceived as more flexible relative to social identities. However, there were no differences in the perceived importance or valence of role, relational, and social identities. While this finding provides initial evidence that identity characteristics are related to the experience and management of conflict, as these were general measures the findings should be interpreted with caution until they are assessed in terms of the conflicting identities themselves.

## General Discussion

Most people have multiple identities. Yet, how these identities are experienced and managed when they conflict is not well understood. In three studies we uncovered new and interesting insights into the nature of identity conflicts, and the experience and management of identity conflicts. To our knowledge these findings are the first to develop a robust measure of identity conflict management strategies as well as the first to find evidence that the types of identities matter for the management though not necessarily the experience of conflict.

### The Nature of Identity Conflicts

In considering the nature of conflict, we found that conflicts between roles, relationships, and life-stages were reported most frequently rather than social identities which tend to figure prominently in the literature on multiple identities and conflict. This highlights the diversity of identity conflicts and suggests that although social identities are important, researchers should also start thinking more broadly about the range of identity conflicts that can be experienced and their implications for individuals’ lives. Although role identities have started to receive more attention (e.g., Hodges and Park, 2013; Rabinovich and Morton, 2016), relational identities have been relatively absent from the literature and require further consideration.

### The Experience of Identity Conflict

In examining the experience of conflict, individuals generally reported similar experiences of conflict between role, relational, and social identities. In particular, the magnitude and growth associated with these conflicts was equivalent across studies. The finding for magnitude in particular is consistent with the idea that experiencing identity conflicts leave individuals similarly torn: the perceived discrepancy associated with these conflicts does not differ whether contending with role, relational, or social identities. This finding is important because it provides further evidence for the use of the BII as a measure of the experience of conflict (Cheng and Lee, 2013) and demonstrates that when any types of multiple identities are implicated in conflict, these conflicts are likely to be felt in similar ways. Growth was also generally experienced in similar ways across different types of conflicts. This finding suggests that when individuals are faced with difficulties such as identity conflict, irrespective of the specific nature of these conflicts, they may be similarly likely to draw on these experiences to learn and change as individuals (e.g., Tedeschi and Calhoun, 2004).

### The Management of Identity Conflict

In considering the management of conflict, individuals reported engaging in a range of strategies, including reconciliation, retreat, and realignment, and we found evidence for the use of these three strategies and a fourth strategy, reflection, to manage these conflicts. All individuals endorsed the use of reconciliation, which suggests that this may reflect the default strategy when facing identity conflicts. This is in line with Nowak et al. (2000) work on the self-system which suggests that the global integration of information is a default process for the self as well as Berry’s work on acculturation, where integration is associated with positive adaptation (Berry et al., 2006; Sam and Berry, 2010). When identities conflict individuals may therefore be motivated to ‘bring their identities back into line’ by engaging in reconciliation. Critically, conflicts between role and relational identities were associated with more use of realignment and retreat, respectively, relative to other types of identities, suggesting that while individuals try to hold onto both identities, roles and relationships may also be differentially shorn as a part of the management process.

### Identity Characteristics and the Experience and Management of Conflict

We also considered whether identity characteristics might help us to understand the experience and management of conflicts. In thinking about why conflicts between multiple roles and multiple relationships are associated with varied management strategies relative to conflicts we found that participants perceived role, relational, and social identities as differing in flexibility but not in importance or valence. Furthermore, the greater perceived flexibility of one role in general, student identity, was associated with the experience and strategic management of conflict. This suggests that when identities are perceived as flexible individuals might ‘feel more’ and believe that they can and/or need to ‘do more’ when they experience identity conflict. Although these relationships are encouraging we recognize that identity characteristics were assessed in general rather than in terms of the specific conflicting identities (e.g., Settles, 2004). However, these initial findings suggest that considering the perceived flexibility of the multiple identities in particular is likely to provide a clearer picture of the differences between roles, relationships, and social categories that might shape the experience and management of conflict.

Taken together, we contend that our measures of the experience and management of identity conflict are robust and provide meaningful insights into the ways that people experience conflict between multiple identities as well as what they might do to manage these conflicts. Yet, these findings also highlight that the experience and management conflicts between different types of identities may not be mirrored across samples. In hindsight, perhaps we should not have expected this to be the case given the subjective nature of identity conflict. Indeed, even though we assigned people to discuss the same conflicts, the nature of these conflicts could differ. Although assessing conflicts between different types of multiple identities helped us in identifying and building robust measures we recognize that moving these ideas forward will likely require a focus on specific types of multiple identities in isolation.

### Limitations and Future Research

Although this research provides initial evidence that experiencing identity conflict is associated with its management and that the types of identities matter for conflict management, these studies may be limited in their generalizability. In particular, the range of conflicts initially reported (i.e., Study 1) may reflect the context in which the data were collected. It is possible that data collected in settings other than university campuses, and with participants other than university students, might elicit very different identity conflicts, and potentially reveal different conflict management strategies. Moreover, university students may have more limited and/or more flexible roles than do older adults. As such, it would be important to explore the nature of role conflicts specifically, and identity conflicts in general, among adults in community samples who may have more fixed role identities, and who may have more experience in managing potential conflicts between their identities. Sample diversity will allow us to consider broader definitions of multiple role, relational, and social identities as well as enabling further tests of the experience of conflict, and the development if necessary, of additional strategies for identity conflict management.

The present findings were also all based on self-reports taken at one moment in time. Given the dynamic process of identity conflict and management, longitudinal studies that track individuals over time to see how the process of conflict unfolds, and whether there is a sequence in which management strategies are used, are recommended. Findings from Study 1 suggest that individuals may also be ‘doing nothing’ or engaging in other neutral strategies that suggest that there might have to be some ‘stew’ time before people act to resolve these identity conflicts. In looking at this process longitudinally, it might be possible to determine whether there are different motivational factors associated with engaging in some strategies rather than others, whether there is a particular time in which this process occurs, and whether there are individual differences in the length of time it takes to resolve conflicts. This type of analysis can offer a clearer picture of the conflict process and can shed light on factors that may be associated with adaptive vs. maladaptive coping with identity conflict.

Finally, with regards to all of these studies, there is a focus on explicit conflict. However, conflict does not always occur explicitly. In fact, the activation of identities is likely an implicit process, with individuals not consciously attending to the cues that elicit these identities (e.g., Sinclair et al., 2006), particularly when contending with less flexible identities. This may explain why conflicts between role identities and relational identities, rather than social identities emerged as the most frequently reported conflicts – it may be easier for individuals to be consciously aware of conflicts arising from roles as they are associated with explicit actions, or between relationships because they can pinpoint disagreements associated with significant others. However, where social identities are concerned, conflicts may be less easy to identify or may be more ambiguous due to the attributions that one can make for conflict leading these types of conflicts to be under-reported. Asking people about their identity conflicts may therefore change the actual experience of these conflicts. Using implicit approaches to investigate identity conflicts (e.g., Hodges and Park, 2013) may provide important insights into the experience and management of conflicts between different types of multiple identities.

## Concluding Remark

Conflict may be inevitable as individuals navigate the multiple identities that help them to make sense of who they are and their social world. Although the present research offers important insights into the experience and management of conflict between multiple identities, these relationships require further scrutiny to understand why and when the types of identities might matter.

## Ethics Statement

This study was carried out in accordance with the recommendations of ‘Human Research Ethics Committee, York University (Canada)’ and ‘Queen Mary University of London Research Ethics Committee’ (United Kingdom) with written informed consent from all subjects. All subjects gave written informed consent in accordance with the Declaration of Helsinki. The protocol was approved by the ‘Human Research Ethics Committee at York University (Canada) (Studies 1–3) and Queen Mary University of London Resarch Ethics Committee (Study 3).’

## Author Contributions

JJ developed the idea, designed the studies, and analyzed and interpreted the data. MH assisted in the development of the idea, study design, and interpretation of the data.

## Conflict of Interest Statement

The authors declare that the research was conducted in the absence of any commercial or financial relationships that could be construed as a potential conflict of interest.

## Footnotes

1 Participants were also asked: when and/or where it occurred, who else was involved, how they felt when they experienced this conflict. For brevity, these descriptive data are not reported here.

2 Three additional factors emerged. However, as these factors did not comprise at least 2 items loading at 0.50 (factors 6 and 7 had no items loading at 0.5 or higher, factor 8 had 1 item loading at 0.5), they were not included in the present analysis. As principal axis factoring (PAF) was used, the variance accounted for was computed by re-running the PAF with the number of selected items (15), and dividing the rotated sums of squared loadings by the number of items in the solution.

3 As not all participants responded to all aspects (e.g., unemployed participants did not complete job/employee ratings) the *n*s differ across the subsequent comparisons.

4 Ethnic minorities might be more likely to experience and manage bi-cultural conflicts, which could explain why this conflict less prominent in the predominantly White sample of Study 3. In fact, several of the White respondents assigned to this condition explained the lack of conflict in similar ways before selecting another conflict (e.g., “I am white and British as are most British people. There is no conflict,” “My ethnic and national identities have never conflicted. America is synonymous with my race”). Accordingly, we re-analyzed the data for Studies 2 and 3 controlling for whether participants were White or non-White. The pattern of findings was almost exactly the same with one exception: In Study 2, the main effect for conflict management strategies became marginal.

5 We conducted an exploratory analysis to determine whether the characteristics differed as a function of the types of identities in general. We conducted three single-factor within subjects ANCOVAs with gender as the covariate for each characteristic. Conflict occurrence and conflict choice were not used as covariates because the ratings of these characteristics were unrelated to the experience or management of conflict. All pairwise comparisons were conducted with Least Significant Difference (LSD): *Flexibility*: A 4 Type of Identity (Student, Employee, Relational, and Social) ANCOVA found that student identity was perceived as more flexible (*M* = 3.61, *SE* = 0.095) relative to relational identities (*M* = 3.27, *SE* = 0.085, *p* = 0.005) or social identities (*M* = 2.95, *SE* = 0.095; *p* < 0.001). Employee identity (*M* = 3.61, *SE* = 0.095) and relational identities were perceived as more flexible than social identities (both *p*s < 0.005). Student and employee identity did not differ from each other in perceived flexibility (*p* = 0.122). *Importance:* A 3 Type of Identities (Role, Relational, and Social) ANCOVA indicated that importance ratings did not differ as a function of the types of identities, *F*(2,346) = 0.39, *p* = 0.68, $\eta_{p}^{2}$ = 0.00. *Valence:* A 3 Type of Identities (Role, Relational, and Social) ANCOVA indicated that valence not differ as a function of the types of identities, *F*(2,346) = 1.74, *p* = 0.18, $\eta_{p}^{2}$ = 0.01. These findings suggest that flexibility might differentiate roles, relationships and social categories from each other.
