# Supplementary material for: Similarly Torn, Differentially Shorn? The Experience and Management of Conflict between Multiple Roles, Relationships, and Social Categories
Source: Front Psychol. 2017 Oct 5;8:1732. doi: 10.3389/fpsyg.2017.01732 (PMC5633912; doi:10.3389/fpsyg.2017.01732)
Supplement: Supplementary file 1 [file Data_Sheet_1.docx]

**Appendix A**

Instructions

*Description of Identity, Conflict, and Identity Conflict*

Individuals can define themselves using a variety of identities. Your identities are those characteristics that shape how you perceive yourself and the world around you. We believe that your identities can be based on a variety of things including the different groups to which you can belong or support (*e.g., race, gender, ethnic groups, political affiliation, sports teams*), the different relationships you may have with other people (*e.g., friend, romantic partner, spouse, co-worker*), the different roles you may play in society (*e.g., student, athlete, daughter/son, volunteer, employee*), or the different life-stages that you find yourself at (*e.g., teenager, adult, senior*).

Because individuals are defined by multiple identities, sometimes *conflict* may occur because of the different identities that make up who you are. Conflict is something that is experienced by most people at some time in their lives. Conflicts between your identities may represent a struggle between different attitudes, beliefs, desires, expectations, morals, norms, obligations, thoughts and/or values that are held by you. For example, you may experience conflict between your identities as an athlete and a student because your obligations to the team may conflict with your ability to get your homework finished *OR* you may experience conflict between your couple identity and family identity because you hold positive attitudes towards your partner, but your parents hold negative attitudes, towards him or her.

Before proceeding to the next page, please take a moment to think about the different identities that are important to you, and the conflicts that you may have experienced between two identities.

**Appendix B**

Rotated Pattern Matrix for Conflict Management Strategies; Principal Axis Factoring with Direct Oblimin Rotation (*Study 2*)

| **Items** | **Factors** | | | | |
| --- | --- | --- | --- | --- | --- |
|  | **Retreat** | **Reconciliation** | **Realignment** | **Reflection** | ***Relinquishment*** |
| I actively avoid situations that cause conflict between these two identities. | .75 |  |  |  |  |
| I try to stay clear of the situations where my two identities might contradict each other. | .62 |  |  |  |  |
| Experiencing conflict between these identities makes me want to withdraw from the situation. | .59 |  |  |  |  |
| I put effort into dividing these identities so they do not conflict. | .56 |  |  |  |  |
| I try to find a middle ground to keep at least some aspects of both identities. |  | .69 |  |  |  |
| I integrate aspects of both identities into who I am |  | .68 |  |  |  |
| I try to balance both of these identities. |  | .63 |  |  |  |
| Rather than 'giving in' to one identity, I try to think of myself in terms of both identities. |  | .56 |  |  |  |
| One of these identities is more important to me than is the other |  |  | .77 |  |  |
| I focus more on one of these identities than on the other |  |  | .58 |  |  |
| I prefer to describe myself in term of one of these identities over the other |  |  | .54 |  |  |
| I express the identity that helps me fit into my environment |  |  |  | .76 |  |
| I choose the identity that helps me fit in with other people |  |  |  | .72 |  |
| *I avoid categorizing myself in terms of either of these identities.* |  |  |  |  | *.54* |
| *I think of myself as an individual, not necessarily expressing any particular identity.* |  |  |  |  | *.51* |

**Appendix C**

Rotated Pattern Matrix for Conflict Management Strategies; Principal Axis Factoring with Direct Oblimin Rotation (*Study 3*)

| **Items** | **Factors** | | | | |
| --- | --- | --- | --- | --- | --- |
|  | **Reconciliation** | **Retreat** | **Reflection** | **Realignment** |  |
| I actively avoid situations that cause conflict between these two identities. |  | .95 |  |  |  |
| I try to stay clear of the situations where my two identities might contradict each other. |  | .72 |  |  |  |
| Experiencing conflict between these identities makes me want to withdraw from the situation. |  | .43 |  |  |  |
| I put effort into dividing these identities so they do not conflict. |  | .49 |  |  |  |
| I find a middle ground to keep at least some aspects of both identities. | .79 |  |  |  |  |
| I integrate aspects of both identities into who I am | .66 |  |  |  |  |
| I try to balance both of these identities. | .83 |  |  |  |  |
| Rather than 'giving in' to one identity, I try to think of myself in terms of both identities. | .57 |  |  |  |  |
| One of these identities is more important to me than is the other |  |  |  | .70 |  |
| I focus more on one of these identities than on the other |  |  |  | .77 |  |
| I prefer to describe myself in term of one of these identities over the other |  |  |  | .60 |  |
| I express the identity that helps me fit into my environment |  |  | .69 |  |  |
| I choose the identity that helps me fit in with other people |  |  | .83 |  |  |
